# Comparative analysis of different biofactories for the production of a major diabetes autoantigen

**DOI:** 10.1007/s11248-013-9749-9

**Published:** 2013-10-20

**Authors:** Linda Avesani, Matilde Merlin, Elisa Gecchele, Stefano Capaldi, Annalisa Brozzetti, Alberto Falorni, Mario Pezzotti

**Affiliations:** 1 Department of Biotechnology, University of Verona, Verona, Italy; 2 Department of Internal Medicine, University of Perugia, Perugia, Italy

**Keywords:** Molecular farming, Recombinant protein production, hGAD65mut, hGAD65, Autoimmune diabetes

## Abstract

The 65-kDa isoform of human glutamic acid decarboxylase (hGAD65) is a major diabetes autoantigen that can be used for the diagnosis and (more recently) the treatment of autoimmune diabetes. We previously reported that a catalytically-inactive version (hGAD65mut) accumulated to tenfold higher levels than its active counterpart in transgenic tobacco plants, providing a safe and less expensive source of the protein compared to mammalian production platforms. Here we show that hGAD65mut is also produced at higher levels than hGAD65 by transient expression in *Nicotiana benthamiana* (using either the pK7WG2 or MagnICON vectors), in insect cells using baculovirus vectors, and in bacterial cells using an inducible-expression system, although the latter system is unsuitable because hGAD65mut accumulates within inclusion bodies. The most productive of these platforms was the MagnICON system, which achieved yields of 78.8 μg/g fresh leaf weight (FLW) but this was substantially less than the best-performing elite transgenic tobacco plants, which reached 114.3 μg/g FLW after six generations of self-crossing. The transgenic system was found to be the most productive and cost-effective although the breeding process took 3 years to complete. The MagnICON system was less productive overall, but generated large amounts of protein in a few days. Both plant-based systems were therefore advantageous over the baculovirus-based production platform in our hands.

**Electronic supplementary material:**

The online version of this article (doi:10.1007/s11248-013-9749-9) contains supplementary material, which is available to authorized users.

## Introduction

Type-1 diabetes (T1D) is a chronic disease caused by the autoimmune destruction of insulin-producing pancreatic β-cells. The incidence of the disease is increasing by approximately 3 % per year and it requires life-long insulin replacement therapy (Aanstoot et al. 2007). The 65-kDa isoform of human glutamic acid decarboxylase (hGAD65), which catalyzes the decarboxylation of glutamate to γ-aminobutyrate (GABA) and CO_2_ (Soghomonian and Martin 1998; Capitani et al. 2003; Gut et al. 2006), is one of the major T1D autoantigens. Autoreactivity against hGAD65 is a valuable marker that can be used both to classify and monitor the progression of the disease (Schmidt et al. 2005). Autoantibodies against GAD65 are considered predictive markers when tested in combination with other disease-specific autoantibodies (Kulmala et al. 1998).

Studies using animal models have shown that exposure to GAD65 may be therapeutic by inducing tolerance (Kaufman et al. 1993; Tisch et al. 1993). Human clinical investigations in recent-onset T1D patients using alum-formulated hGAD65 therefore considered the safety and efficacy of a treatment regimen consisting of prime and boost injections with different doses of the protein (Lernmark and Agardh 2005). Although these studies showed that treatment was safe, the efficacy data were equivocal suggesting that inducing tolerance in humans remains a challenge (Wherrett et al. 2011; Ludvigsson et al. 2012). A further trial, involving genetically-predisposed children and young adults with multiple islet cell autoantibodies, is currently exploring the ability of alum-formulated hGAD65 to prevent the onset of disease (NCT01122446). Future strategies may include combination therapies coupling immunosuppressive agents with one or more autoantigens (Larsson and Lernmark 2011).

The large-scale production of full-length recombinant hGAD65 currently involves the use of either insect cells (Moody et al. 1995) or methylotrophic yeast (Raymond et al. 1998) both of which are expensive and vulnerable to contamination. The growing demand for high-quality hGAD65 for diagnostic and therapeutic applications means that alternative platforms are required to ensure there is sufficient production capacity in the future. The production of hGAD65 in plants was previously reported (Avesani et al. 2003, 2007, 2010; Ma et al. 2004; Morandini et al. 2011) including a catalytically-inactive derivative autoantigen (hGAD65mut) that retains its immunogenic properties and accumulates to tenfold higher levels than its wild-type counterpart (Avesani et al. 2010). We hypothesized that the wild-type version of hGAD65 interferes with plant cell metabolism to suppress its own synthesis, whereas the catalytically-inactive version escapes such feedback and accumulates to higher levels.

The hGAD65mut mutant was generated by substituting the lysine residue that binds the co-factor pyridoxal 5′-phosphate (PLP) with an arginine residue (K396R). The mutant protein has been produced in a cell-free transcription and translation system (Hampe et al. 2001) and in transgenic tobacco plants (Avesani et al. 2010) and in each case binds autoantibodies from the sera of T1D patients.

We developed a hypothesis that hGAD65mut is intrinsically more suitable for heterologous expression than hGAD65, and should accumulate to higher levels than the wild-type protein in different production platforms such as bacteria, insect cells and *Nicotiana benthamiana* plants. We therefore tested a commercial *E. coli* platform containing an inducible vector, *Spodoptera frugiperda* cells infected with the Baculodirect Expression System (Life Technologies) and two transient expression vectors (pK7WG2 and the MagnICON system) in *N. benthamiana* plants. These systems were compared with the best-performing stable transgenic tobacco lines we previously reported, which have been improved by several generations of conventional breeding starting with the T1 generation.

## Materials and methods

### Self-pollination of elite tobacco plants

The flowers of hGAD65mut transgenic plants were bagged before blooming to prevent cross-pollination, and the bags were collected and stored after blooming, fruit ripening and seed drying. Starting from the best-performing hGAD65mut T1 plants, the dried seeds were sown to produce subsequent generations of transgenic tobacco plants up to the T6 generation.

### Construction of plant expression vectors

The pK7WG2.G65 and pK7WG2.G65mut vectors were constructed as previously described (Avesani et al. 2010). To obtain final TMV 3′ modules carrying the genes of interest, hGAD65 and hGAD65mut were amplified by PCR using forward (5′-TTT GGT CTC AAG GTA TGG CAT CTC CGG GCT CTG GCT TTT GG-3′) and reverse (5′-TTT GGT CTC AAA GCT TAT TAT AAA TCT TGT CCA AGG CGT TC-3′) primers and inserted in the pGEM-T Easy vector (Promega, Madison, WI). The pGEM.G65 and pGEM.G65mut vectors were used as entry clones for recombination with the TMV 3′ module pICH31070, as described by Engler et al. (2008).

### Transient expression in *N. benthamiana*

The pK7WG2.G65 and pK7WG2.G65mut vectors were introduced into *Agrobacterium tumefaciens* strain EHA105. The bacteria were cultivated for 2 days in YEB medium containing 50 μg/ml rifampicin, 300 μg/ml streptomycin and 100 μg/ml spectinomycin, pelleted by centrifugation at 4,000×*g* and resuspended in infiltration buffer (10 mM MES, 10 mM MgCl_2_, 100 μM acetosyringone, pH 5.6) to an OD_600_ of 0.9. Following incubation for 3 h at room temperature, bacterial suspensions were syringe infiltrated into 5–6-week-old *N. benthamiana* plants, using three leaves per plant (one biological replicate). Leaves were infiltrated with the pK7WG2 vector carrying the *gfp* marker gene as a negative control. The leaves of each biological replicate were sampled 2 days post-infiltration (dpi).

For TMV-based expression, pICH31070.G65 and pICH31070.G65mut (3′ modules), pICH20111 (5′ module) and pICH14011 (integrase module) were introduced into *A. tumefaciens* strain GV3101. The bacteria were seeded into LB medium containing 50 μg/ml rifampicin and 50 μg/ml kanamycin (3′ modules) or 50 μg/ml carbenicillin (integrase and 5′ modules). Overnight bacterial cultures were collected by centrifugation at 4,000×*g* and resuspended in two volumes of 10 mM MES (pH 5.5) and 10 mM MgSO_4_. Equal volumes of the hGAD65 or hGAD65mut 3′ module, 5′ module and integrase module suspensions were mixed and used to infiltrate the leaves of 5–6-week-old *N. benthamiana* plants, with each biological replicate comprising a pool of three infiltrated leaves, sampled at 4 dpi. A mixture of the 5′-module and integrase-module suspensions was used as a negative control. The plants were grown in an enclosed chamber at 25/22 °C day/night temperature with a 16-h photoperiod.

### Expression using the baculovirus/insect cell system

Recombinant baculovirus DNA was obtained by LR recombination between pENTR™/D-TOPO.G65 or pENTR™/D-TOPO.G65mut (Avesani et al. 2010) and the linearized viral DNA. Sf9 cells were seeded into 6-well plates (8 × 10^5^ cells per well) and washed twice with 2 ml of non-supplemented Grace’s Insect Medium (Life Technologies, Paisley, UK). The medium was removed and replaced drop-wise with the transfection mixture (5 μl LR recombination reaction, 6 μl Celfectin solution and 200 μl non-supplemented Grace’s Insect Medium). The plates were incubated at 27 °C for 5 h before the transfection mixture was removed and replaced with 2 ml fresh Sf-900 medium (Life Technologies, Paisley, UK) supplemented with 10 % fetal bovine serum, 10 μg/ml gentamicin and 100 μM ganciclovir for the selection of recombinant baculovirus clones. After incubation for 96 h at 27 °C, the medium (V1 viral stock) was collected, centrifuged at 4,000×*g* to remove cells and large debris, and stored in the dark at 4 °C. High-titer V2 viral stock was generated by seeding 1 × 10^6^ Sf9 cells per well in 2.5 ml Sf-900 medium containing 10 % fetal bovine serum, 10 μg/ml gentamicin and 100 μM ganciclovir, and infecting with 100 μl of the V1 stock. The cells were incubated for 3 days at 27 °C, the medium was collected and centrifuged at 4,000×*g*, and the supernatant (V2 stock) was stored at 4 °C.

### Expression in bacterial cells

The Gateway destination vector pDEST17 (Life Technologies, Paisley, UK) was isolated from *E. coli* DB3.1 cells (Life Technologies, Paisley, UK) and used for LR recombination with the entry vectors pENTR™/D-TOPO.G65 and pENTR™/D-TOPO.G65mut (Avesani et al. 2010), yielding pDEST17.G65 and pDEST17.G65mut, respectively. The pDEST17.CmR vector carrying a chloramphenicol-resistance gene was used as a negative control. The three expression vectors were independently transferred to electrocompetent *E. coli* BL21 (DE3) cells (Novagen, Madison, WI) and individual colonies were cultured overnight at 37 °C in ampicillin-containing LB medium. The culture was then diluted 1:100 with LB medium and incubated at 37 °C for 1–6 h until the OD_600_ reached 0.8. Recombinant protein expression was induced with 1 mM isopropyl-β-D-thiogalactopyranoside (IPTG; Sigma-Aldrich, St. Louis, MO) and the culture was incubated at 37 °C for 3 h before the cells were collected by centrifugation at 4,000×*g* and stored at −80 °C prior to protein extraction.

### Analysis of recombinant protein expression

Total soluble proteins were extracted from plant tissues by grinding to fine powder under liquid nitrogen and homogenizing in extraction buffer (40 mM HEPES pH 7.9, 5 mM DTT, 1.5 % CHAPS) supplemented with Protease Inhibitor Cocktail (Sigma-Aldrich, St. Louis, MO). Bacterial cells were collected by centrifugation at 4,000×*g* and resuspended in half the culture volume of TBS (20 mM Tris–HCl pH 7.4, 500 mM NaCl) supplemented with 1 mM phenylmethanesulfonylfluoride (PMSF; Sigma-Aldrich, St. Louis, MO) then sonicated on ice three times for 40 s at half power. The lysate was clarified by centrifugation at 14,000×*g* for 20 min at 4 °C. The supernatant and pellet were stored separately at −80 °C. The inclusion bodies were solubilized with 6 M urea and stored at −80 °C. Infected insect cells were collected by centrifugation at 3,000×*g* for 5 min, washed with 1 ml PBS, resuspended in 200 μl lysis buffer (20 mM Tris/HCl pH 8.0, 0.5 M NaCl, 10 mM imidazole, 3 mM β-mercaptoethanol and 1 % Tween-20) and incubated on ice for 30 min. The solubilized cells were centrifuged at 14,000×*g* at 4 °C for 20 min and the soluble fractions were collected and stored at −80 °C.

Radioimmunoassays (RIAs) were carried out using hGAD65 autoantibody-positive serum from a T1D patient and ^125^I-GAD65 (RSR, Cardiff, UK) as a tracer (Falorni et al. 1994). Commercial recombinant human GAD65 (rhGAD65) produced in the baculovirus expression system (Diamyd, Karlavagen, SE) was used as positive control. Non-transformed controls were analyzed in parallel to exclude potential negative effects caused by the buffer and host components during the detection procedure.

The protein samples were separated by SDS-PAGE on a 10 % polyacrylamide gel and transferred to a nitrocellulose membrane by electroblotting. Proteins were detected using the GC3108 (IgG1) monoclonal antibody (Biomol International, Farmingdale, NY) as previously described (Avesani et al. 2003).

## Results

### Stable expression of hGAD65 and hGAD65mut in tobacco and the establishment of a homogeneous transgenic tobacco platform for hGAD65mut

We previously reported the expression of hGAD65 and hGAD65mut in transgenic tobacco plants (Avesani et al. 2010). As expected, the recombinant protein levels varied significantly among independently-transformed lines, probably reflecting the position effects associated with random transgene insertion (Krysan et al. 2002). We compared the accumulation of the two proteins in T1 transgenic lines by selecting the three best-performing individuals (elite lines) evaluated by RIA using GAD65 autoantibody-positive serum (Table 1). This comparison showed that the average yield in the elite hGAD65mut pre-flowering lines was 143.6 μg/g FLW, 13-fold higher than the 10.5 μg/g FLW average yield in the hGAD65 elite lines (Table 1). The observed difference was statistically significant (Student’s *t* test, *p* < 0.01).

**Table 1:** Yields of the recombinant protein in the different platforms analyzed

System	Molocule	[GAD] (μg/ml)	[GAD]	[hGAD65mut]/[hGAD65]
“Elite” T1	hGAD65	3.5 ± 0.9	10.5 ± 2.6 μg/g FLW	13.7 ± 4.5
	hGAD65mut	47.9 ± 10.6	143.6 ± 31.7 μg/g FLW	
Transient	hGAD65	1.4 ± 0.4	4.3 ± 1.3 μg/g FLW	15.8 ± 4.8
	hGAD65mut	22.6 ± 0.9	67.8 ± 2.7 μg/g FLW	
MagnICON	hGAD65	9.0 ± 0.2	26.9 ± 0.7 μg/g FLW	2.9 ± 0.7
	hGAD65mut	26.3 ± 5.9	78.8 ± 17.8 μg/g FLW	
Baculo/insect	hGAD65	77.4 ± 7.4	7.7 ± 0.7 μg/ml colture medium	1.5 ± 0.2
	hGAD65mut	117.5 ± 7.7	11.8 ± 0.8 μg/ml colture medium	

**Fig. 1:**
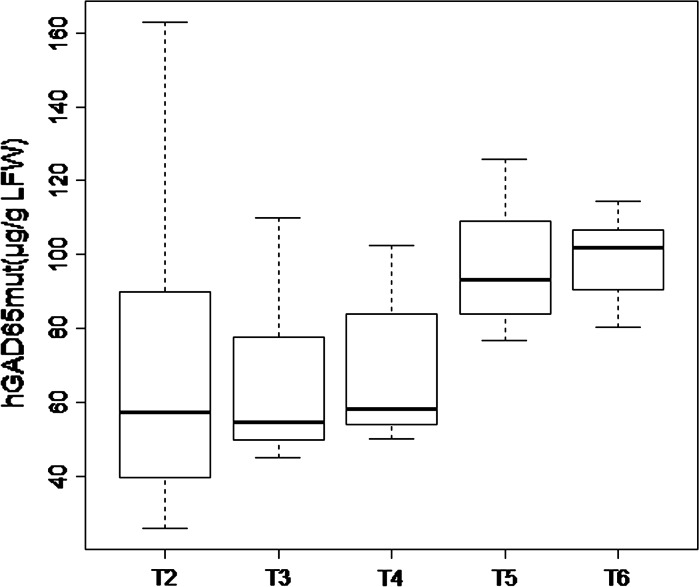
Accumulation of hGAD65mut over several generations derived from the best-performing hGAD65mut T1 transgenic tobacco plant. *Boxplot* representation of hGAD65mut levels in μg/g FLW calculated from radioimmunoassay data

We developed a homogeneous production platform by self-crossing the best-performing T1 hGAD65mut transgenic plant and repeating the self-crossing over several generations, checking the performance in each generation by RIA until no further improvement was achieved (data not shown). The average yield increased from 68.9 μg/g FLW in T2 to 99.1 μg/g FLW in T6 (Fig. 1 and Online Resource 1). During the selection process, the standard deviation in the expression level declined from 40.1 in T2 to 11.33 in T6 (Online Resource 1).

### Transient expression of hGAD65 and hGAD65mut in *N. benthamiana* using pK7WG2

The hGAD65 and hGAD65mut sequences were cloned separately in pK7WG2, which was previously used for the stable transformation of tobacco (Karimi et al. 2002). The resulting vectors pK7WG2.G65 and pK7WG2.G65mut were separately introduced into *A. tumefaciens* and infiltrated into three leaves on three different *N. benthamiana* plants. Time-course analysis showed that protein accumulation peaked at 2 dpi (Online Resource 2). Therefore, the leaves were harvested 2 dpi and protein extracts were analyzed by RIA. The average expression level of hGAD65mut was 67.8 μg/g FLW, which was approximately 16-fold higher than hGAD65 at 4.3 μg/g FLW (Table 1). This was a statistically significant difference (Student’s *t* test, *p* < 0.01) and the trend matched our observations of the elite transgenic tobacco lines (Table 1).

**Fig. 2:**
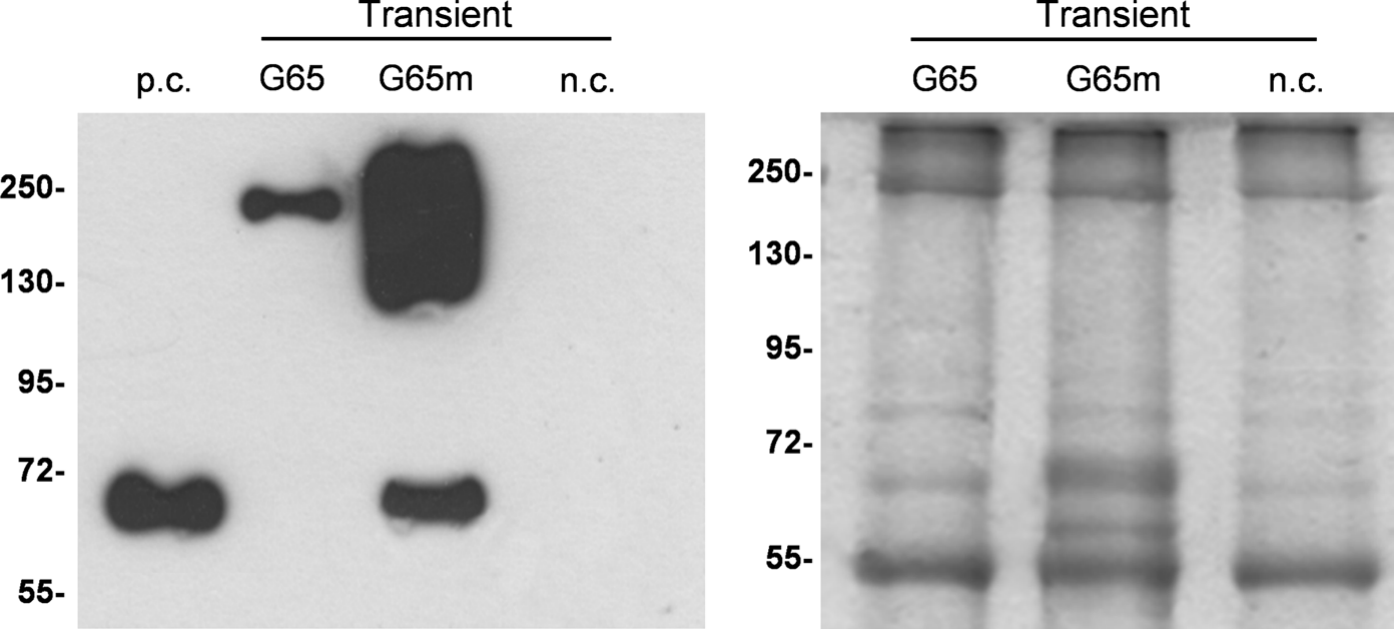
Transient expression of hGAD65 and hGAD65mut in *Nicotiana benthamiana* plants using vector pK7WG2. *Left panel* western blot of hGAD65 (G65) and hGAD65mut (G65m) in leaf extracts (5 μg TSP per lane) detected using the GC3108 antibody. *Right panel* loading control stained with Coomassie Brilliant Blue. *Numbers* indicate the molecular mass markers in kDa. *n.c.* negative control, leaves infiltrated with the pK7WG2 vector carrying the *gfp* marker gene; *p.c.* positive control, 10 ng of commercial rhGAD65-His_6_ produced in the baculovirus/insect cell system

Extracts from the best-performing *N. benthamiana* biological replicates for each construct were investigated in more detail by western blot (Fig. 2). Extracts from leaf pools expressing each construct revealed a major band with an apparent molecular weight >65 kDa, probably reflecting the presence of protein aggregates. The 65-kDa polypeptide, corresponding to the monomeric form of the protein, was only detected in extracts from plants expressing hGAD65mut, although this was more likely to reflect the greater abundance of the protein *per se* in these plants rather than the relatively greater abundance of the monomeric form compared to aggregates. In support of this conclusion, we found that protein aggregates were also more abundant than the monomeric form in western blots of transgenic plants (data not shown). As expected, the anti-GAD monoclonal antibody did not recognize endogenous tobacco proteins.

### MagnICON expression of hGAD65 and hGAD65mut in *N. benthamiana*

The hGAD65 and hGAD65mut sequences were also transiently expressed using the MagnICON deconstructed tobacco mosaic virus (TMV) system. The sequences were cloned separately in the TMV 3′-module pICH31070 (Marillonnet et al. 2004). The final 3′-modules, the pICH20111 5′-module and the pICH14011 integrase-module, were introduced into *A. tumefaciens* separately, and mixed suspensions were used for the agroinfiltration of three leaves from three different *N. benthamiana* plants. Based on previously-determined time-course data (Online Resource 3), the infiltrated leaves were collected 4 dpi.

**Fig. 3:**
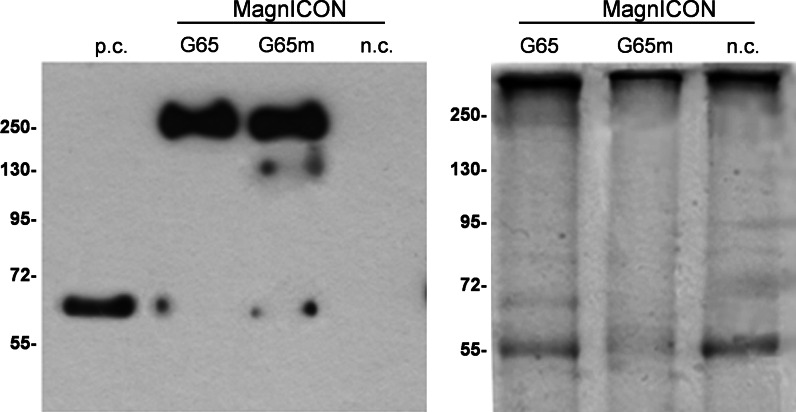
Transient expression of hGAD65 and hGAD65mut in *Nicotiana benthamiana* plants using MagnICON vectors. *Left panel* western blot of hGAD65 (G65) and hGAD65mut (G65m) in leaf extracts (1 μg TSP per lane) detected using the GC3108 antibody. *Right panel* loading control stained with Coomassie Brilliant Blue. *Numbers* indicate the molecular mass markers in kDa. *n.c.* negative control, plant infiltrated with the pICH20111 5′-module and pICH14011 integrase-module; *p.c.* positive control, 10 ng of commercial rhGAD65-His_6_ produced in the baculovirus/insect cell system

The accumulation of immunoreactive recombinant protein was measured by RIA revealing that hGAD65mut accumulated to 78.8 μg/g FLW, almost threefold higher than hGAD65 at 26.8 μg/g FLW, representing a statistically significant difference (Student’s *t* test, *p* < 0.01) albeit less than the difference in the accumulation of the two recombinant proteins observed with the pK7WG2 transient expression approach (Table 1). Western blots of leaf protein extracts from the two best-performing plants confirmed the RIA results, indicating a slight difference between the two recombinant proteins that could be inferred only after normalizing the specific signals with the total soluble proteins stained in the reference gel with Coomassie Brilliant Blue (Fig. 3). As above, both hGAD65 and hGAD65mut predominantly comprised aggregates, whereas the monomeric forms of the proteins appeared as minor components.

### Expression of recombinant hGAD65 and hGAD65mut using baculovirus vectors

**Fig. 4:**
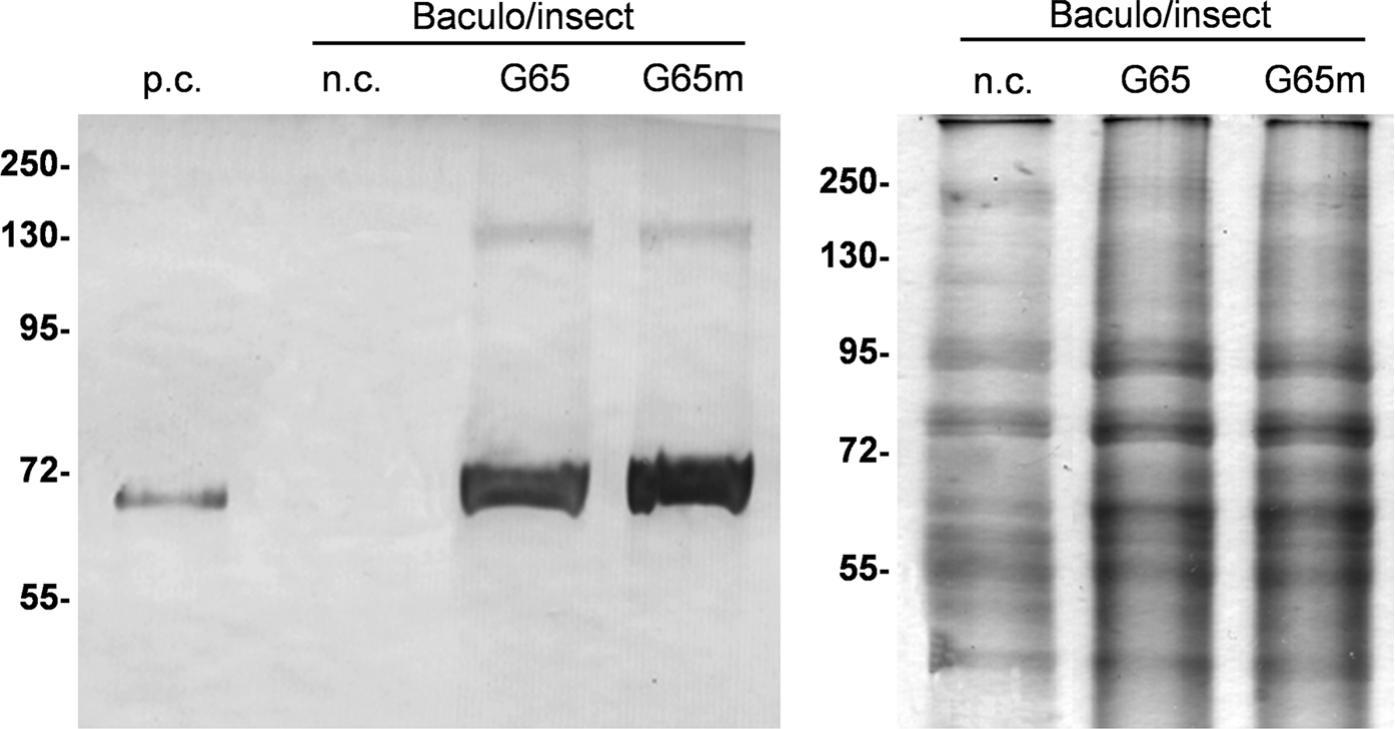
Expression of hGAD65 and hGAD65mut using the baculovirus/insect cell platform. *Left panel* western blot of hGAD65 (G65) and hGAD65mut (G65m) in cell extracts (15 μg TSP per lane) detected using the GC3108 antibody. *Right panel* loading control stained with Coomassie Brilliant Blue. *Numbers* indicate the molecular mass markers in kDa. *n.c.* negative control, non-transformed insect cell extracts; *p.c.* positive control, 10 ng of commercial rhGAD65-His_6_ produced in the baculovirus/insect cell system

Baculovirus vectors containing the hGAD65mut and hGAD65 sequences with a C-terminal His_6_ tag were expressed in adherent Sf9 cell cultures. V1 and V2 high-titer stocks were prepared and the optimal viral stock (Online Resource 4) and time-course kinetics were identified for comparative purposes. Each experiment was carried out in triplicate. RIA analysis revealed a statistically significant difference (Student’s *t* test, *p* < 0.01) in the accumulation of hGAD65mut (11.7 ± 0.8 μg/ml culture medium) and hGAD65 (7.7 ± 0.7 μg/ml culture medium), as shown in Table 1. This difference was difficult to visualize in western blots of extracts from the best-performing cultures (Fig. 4). The anti-GAD antibody did not recognize endogenous insect proteins but primarily detected a specific band migrating at the predicted molecular weight of the monomeric form of the recombinant proteins. With the exception of a weaker 130-kDa band presumably representing a protein dimer, the insect cell cultures were conspicuous by the absence of multimeric aggregates that were most abundant in the plant extracts.

### Expression of hGAD65 and hGAD65mut in *E. coli*

The hGAD65 and hGAD65mut sequences were individually cloned in the Gateway destination vector pDEST17, which allows the induction of transcription with IPTG (Belfield et al. 2007). A chloramphenicol-resistance gene in the same vector was used as a negative control. The three resulting vectors (pDEST17.G65, pDEST17.G65mut and pDEST17.CmR) were introduced into *E. coli* BL21 cells. Individually, the expression of hGAD65mut and hGAD65 was induced in triplicate cultures.

**Fig. 5:**
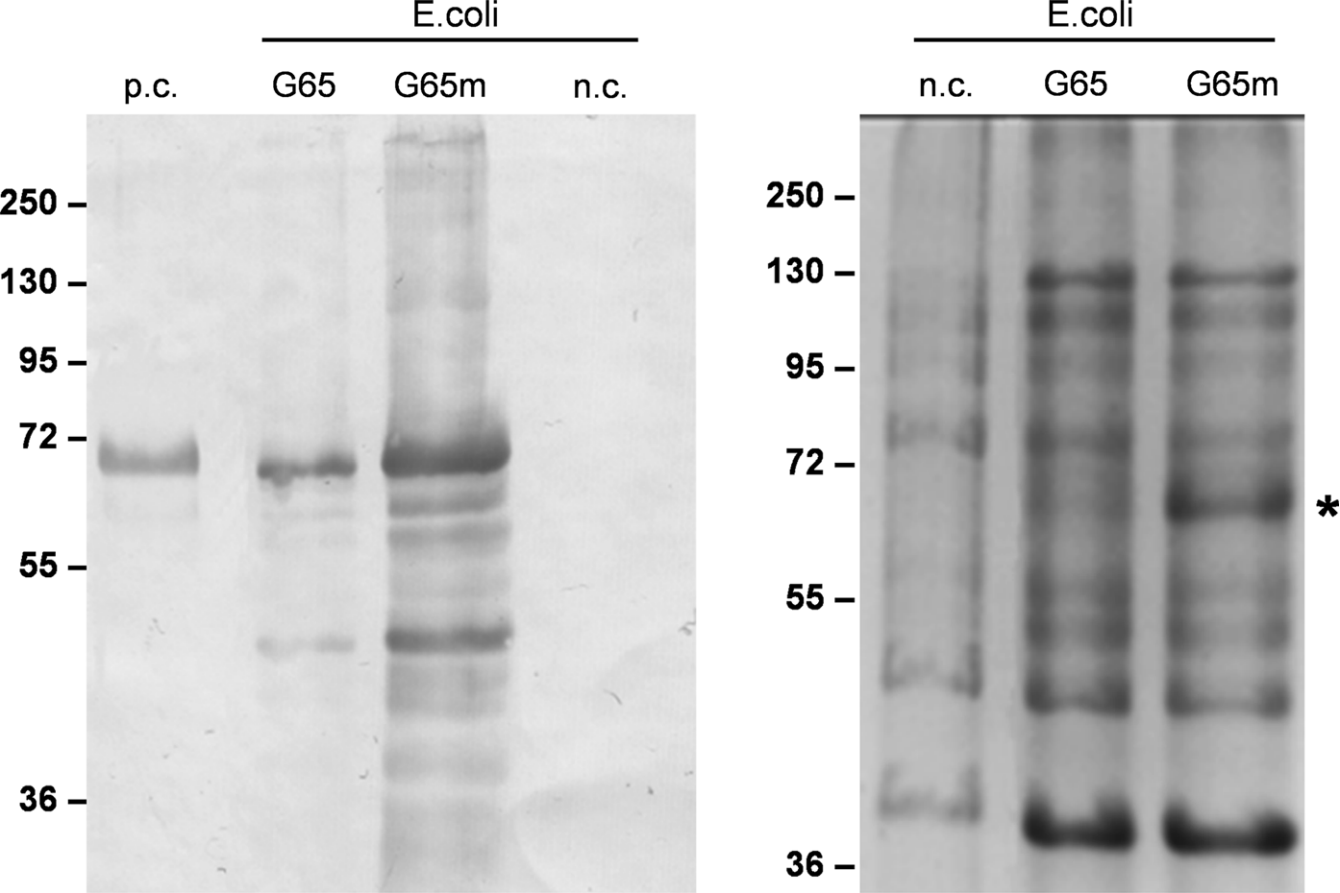
Expression of hGAD65 and hGAD65mut using the *E. coli* inducible expression platform. *Left panel* western blot of hGAD65 (G65) and hGAD65mut (G65m) in cell extracts (2 μg TSP per lane) detected using the GC3108 antibody. *Right panel* loading control stained with Coomassie Brilliant Blue, with *asterisk* indicating the band corresponding to hGAD65mut. *Numbers* indicate the molecular mass markers in kDa. *n.c.* negative control, bacterial cell transformed with the pDest17 ‘empty’ vector; *p.c.* positive control, 15 ng of commercial rhGAD65-His_6_ produced in the baculovirus/insect cell system

Western blots indicated that both hGAD65mut and hGAD65 accumulated in the insoluble fraction (data not shown). We tested several strategies to solubilize the recombinant proteins but only the use of a strong denaturing agent such as 6 M urea was successful (data not shown). Urea interferes with RIA analysis thus preventing the accurate quantification of the recombinant proteins. The western blots confirmed that hGAD65mut accumulated to higher levels than hGAD65 (Fig. 5) but also revealed the presence of polypeptides with a lower molecular mass than expected, suggesting the proteins were degraded or suffered premature translational termination events. The solubility of recombinant proteins produced in *E. coli* can be improved by culturing the cells at lower temperatures (Hunt 2005) but we found that low-temperature cultivation at 15 or 20 °C had no impact on the yield of either hGAD65 or hGAD65mut (Online Resource 5).

## Discussion

We previously reported the expression of hGAD65 and hGAD65mut in stably-transformed tobacco plants (Avesani et al. 2010). The yield of the inactive mutant protein was up to 2.2 % total soluble protein (TSP), which was more than tenfold higher than ever achieved for the wild-type protein. We reasoned that the enzymatic activity of hGAD65 prevented high-level accumulation by suppressing its own synthesis, whereas the inactive version was unaffected by such feedback. Here, we investigated whether this trend was conserved in other expression systems, i.e. transient expression with standard and MagnICON vectors in *N. benthamiana*, inducible expression in *E. coli* and transduction with baculovirus vectors in insect cells. We compared the performance of these expression platforms in terms of recombinant protein yield. We used the original human sequences in all experiments, i.e. the constructs were not optimized for the different platforms.

We began by testing the previously-reported T1 transgenic tobacco plants expressing hGAD65mut and hGAD65 (Avesani et al. 2010). We found that the average yields in the three best-performing elite lines were 143.6 μg/g FLW for hGAD65mut and 10.5 μg/g FLW for hGAD65, which was a tenfold difference. Stable transformation is advantageous because predictable expression levels can be achieved in the offspring of a well-characterized transgenic event, which can give rise to a large population of homogeneous transgenic plants. Hence, the best-performing hGAD65mut elite lines were self-crossed for several generations until recombinant protein yields were homogeneous, probably reflecting homozygosity at the transgenic loci. After six generations of selfing taking more than 3 years, the final average yield of hGAD65mut in the most productive plants was 114.3 μg/g FLW.

In contrast to stable transformation, transient expression in *N. benthamiana* can achieve high yields over relatively short timescales, although there can be significant variation (Voinnet et al. 2003; Chiera et al. 2008; Conley et al. 2011). A high-throughput platform for transient expression in tobacco has also been proposed (Piotrzkowski et al. 2012), consisting of a leaf-disc based infiltration approach that allows different traits to be compared simultaneously on a small scale. We carried out transient expression using the vector previously used to generate transgenic plants (pK7WG2) and observed a similar fold difference in the expression levels of catalytically active hGAD65 and inactive hGAD65mut in both systems. The overall average yield of both proteins was significantly higher in the stably-transformed plants (Student’s *t* test, *p* < 0.05 for both proteins), probably reflecting the benefits of multiple rounds of selection to isolate those plants with the genomic background most favorable for strong transgene expression. Interestingly, the transient expression levels we observed were less variable than the stable expression levels among the T1 transgenic plants, showing it was quicker and easier to generate a relatively homogeneous population without selection, reflecting the presence of hundreds of active but non-integrated copies of the transgene for a few days after agroinfiltration (Kapila et al. 1997). The agreement between the transient and stable expression data in terms of the fold difference between hGAD65 and hGAD65mut suggests that transient expression in *N. benthamiana* gives a reliable forecast of the best-performing transgenic tobacco plants, as previously observed (Conley et al. 2011).

We also tested the MagnICON transient expression platform which is based on deconstructed viral vectors (Gleba et al. 2007) and can achieve yields of up to 4 mg/g FLW (Marillonnet et al. 2005). However, agroinfiltrated plants expressing the MagnICON hGAD65mut vector were significantly less productive than the elite transgenic tobacco lines (Student’s *t* test, *p* < 0.05) and there was no significant difference between the transient expression levels achieved with the MagnICON platform and the standard expression vector pK7WG2 (Student’s *t* test, *p* > 0.05). In contrast, the MagnICON platform significantly outperformed both pK7WG2 transient expression and the transgenic plants in the case of hGAD65 (Student’s *t* test, *p* < 0.01 in both cases). The fold difference in hGAD65mut and hGAD65 expression was therefore lower in the MagnICON platform compared to both pK7WG2 transient expression and the transgenic plants. We observed signs of toxicity (such as premature leaf senescence) when either hGAD65mut or hGAD65 were expressed, as previously reported for other recombinant proteins (Pinkhasov et al. 2011; Nausch et al. 2012). This may explain the lower yields we observed compared to the potential of the system, which can achieve recombinant protein yields of up to 80 % TSP (Marillonnet et al. 2004).

To explain these data, we propose that hGAD65mut reaches a threshold level in the transgenic plants which is determined by the inherent stability of the protein in the plant cell environment, based on its intrinsic physicochemical properties. This cannot be overcome using the MagnICON system. In contrast, the accumulation of hGAD65 in the transgenic plants is inhibited at a much lower level because of the hypothesized feedback mechanism discussed above, which is determined by its catalytic activity. When pK7WG2 is used for transient expression, we propose that the same feedback mechanism kicks in. However, it is possible that the MagnICON can overcome this feedback because of the rapid and high-level expression, allowing large amounts of protein to accumulate before any impact on plant metabolism takes effect.

In addition to the plant-based platforms, we also expressed hGAD65mut and hGAD65 in insect cells using baculovirus vectors, since hGAD65 produced in this system has recently been used for a phase III clinical trial testing the preservation of β-cell function in patients with recent-onset T1D (Ludvigsson et al. 2012). As in plants, we found that hGAD65mut accumulated to a higher level than hGAD65, but the fold difference was the lowest among the platforms we tested, suggesting that hGAD65 is less toxic to insect cells than plant cells.

It has previously been shown that hGAD65 forms inclusion bodies when expressed in *E. coli* (Mauch et al. 1993) so that laborious solubilization and refolding are required to achieve the native conformation (Franke et al. 1988). We therefore expressed hGAD65 and hGAD65mut using an inducible system suitable for protein overexpression (pDEST17/BL2.1) exploiting different growing temperatures to optimize performance. We focused on low-temperature cultivation because this reduces the hydrophobic interactions that are known to promote the formation of inclusion bodies, and in this way we aimed to improve the solubility of the recombinant proteins and encourage efficient folding (Niiranen et al. 2007). Even with the benefits of this platform, we found that both recombinant proteins formed insoluble aggregates under all the conditions we tested. Solubilization of the aggregates using strong denaturing agents suggested that hGAD65mut accumulated to higher levels than hGAD65, but this system is clearly unsuitable for the large-scale production of immunogenic proteins. However, the higher accumulation of hGAD65mut compared to hGAD65 confirms that the catalytic activity of the recombinant protein hampers its accumulation in bacterial cells. Endogenous bacterial GAD is thought to control the acidification of the cytosol so it is likely that the recombinant protein disrupts this process (Capitani et al. 2003).

Overall, our data indicate that hGAD65mut accumulates to a higher level than hGAD65 in all the platforms we tested, although the fold difference is platform-dependent. This is likely to reflect a universal feedback mechanism in which GAD65 enzyme activity interferes with the metabolic processes responsible for its own synthesis, whereas the catalytically inactive form escapes such feedback and accumulates to higher levels (Avesani et al. 2010).

Finally, we selected the best-performing plant system, i.e. the elite transgenic plant line expressing hGAD65mut at the highest level, and compared it in terms of yield and cost with the commercial baculovirus platform used to produce hGAD65. Assuming similar costs for developing the two systems and ignoring personnel costs, we estimated that the production costs for 1 g of recombinant protein using the baculovirus system could reach 700 euro (including conservative costs for the media required to grow 9 l of insect cells) whereas the equivalent cost in plants was substantially lower, at less than 5 euro (including the cost of soil to grow 60 tobacco plants). The costs associated with sterile cell cultures are much higher than the costs associated with growing plants, so even if downstream processing is more difficult and expensive in the case of plants, the baculovirus system remains much more costly overall. It is also notable that plants are much more scalable than cultured cells, so it is clear that transgenic plants offer a significant advantage in terms of overall production and costs even if insect cells have a greater intrinsic productivity per unit of biomass.

## Electronic supplementary material

Below is the link to the electronic supplementary material.

**Online Resource 1 (Table)** Mean, maximum and minimum accumulation values are reported as µg hGAD65mut/g FLW calculated from radioimmunoassay data, and corresponding standard deviations. (TIFF 4760 kb)

**Online Resource 2 (Figure)** Transient expression of hGAD65mut in *Nicotiana benthamiana* plants using the pK7WG2 vector. Samples were collected daily from the 1-5 days post infiltration (dpi) (lanes 1-5). **First panel**, western blot of hGAD65mut (G65m) in leaf extracts (2.5 µg TSP per lane) detected using the GC3108 antibody. **Second panel,** loading control stained with Coomassie Brilliant Blue. Numbers indicate the molecular mass markers in kDa. n.c.= negative control, plants infiltrated with the pK7WG2 vector carrying the *gfp* marker gene; p.c.=positive control, 10 ng of commercial rhGAD65-His_6_ produced in the baculovirus/insect cell system. (TIFF 14749 kb)

**Online Resource 3 (Figure)** Transient expression of hGAD65mut in *Nicotiana benthamiana* plants using MagnICON vectors. Samples were collected daily from 1–8 days post infiltration (dpi) (lanes 1–8). **First panel**, western blot of hGAD65mut (G65m) in leaf extracts (5 µg TSP per lane) detected using the GC3108 antibody. **Second panel,** loading control stained with Coomassie Brilliant Blue. Numbers indicate the molecular mass markers in kDa. n.c. = negative control, plant infiltrated with the pICH20111 5’-module and pICH14011 integrase-module; p.c. = positive control, 10 ng of commercial rhGAD65-His_6_ produced in the baculovirus/insect cell system. (TIFF 12732 kb)

**Online Resource 4 (Figure)** Expression of hGAD65mut using the baculovirus/insect cell platform. The following viral stock volumes were tested: 5, 25 and 50 µl. **Left panel,** western blot of hGAD65mut (G65m) in cell extracts (5 µg TSP per lane). **Right panel,** loading control stained with Coomassie Brilliant Blue. Numbers indicate the molecular mass markers in kDa. n.c. = negative control, extract of non-transformed insect cells; p.c. = positive control, 10 ng of commercial rhGAD65-His_6_ produced in the baculovirus/insect cell system. (TIFF 8599 kb)

**Online Resource 5 (Figure)** Expression of hGAD65mut using the *Escherichia coli* inducible expression platform. Bacterial cells were grown at 15°C or at 20°C. **Upper panel,** western blot of hGAD65mut in cell extracts (2 µg TSP per lane). **Lower panel**, loading control stained with Coomassie Brilliant Blue. Numbers indicate the molecular mass markers in kDa. n.c. = negative control, bacterial cells transformed with the pDEST17 ‘empty’ vector; p.c. = positive control, 15 ng of commercial rhGAD65-His_6_ produced in the baculovirus/insect cell system; T: Total samples; S1: Supernatant after sonication and centrifugation; S2 and P: Supernatant and pellet after centrifugation of the sample solubilized in urea-containing buffer. (TIFF 23553 kb)
