# Supplementary figures and images for: Comparative analysis of different biofactories for the production of a major diabetes autoantigen
Source: Transgenic Res. 2013 Oct 20;23(2):281–91. doi: 10.1007/s11248-013-9749-9 (PMC3951962; doi:10.1007/s11248-013-9749-9)

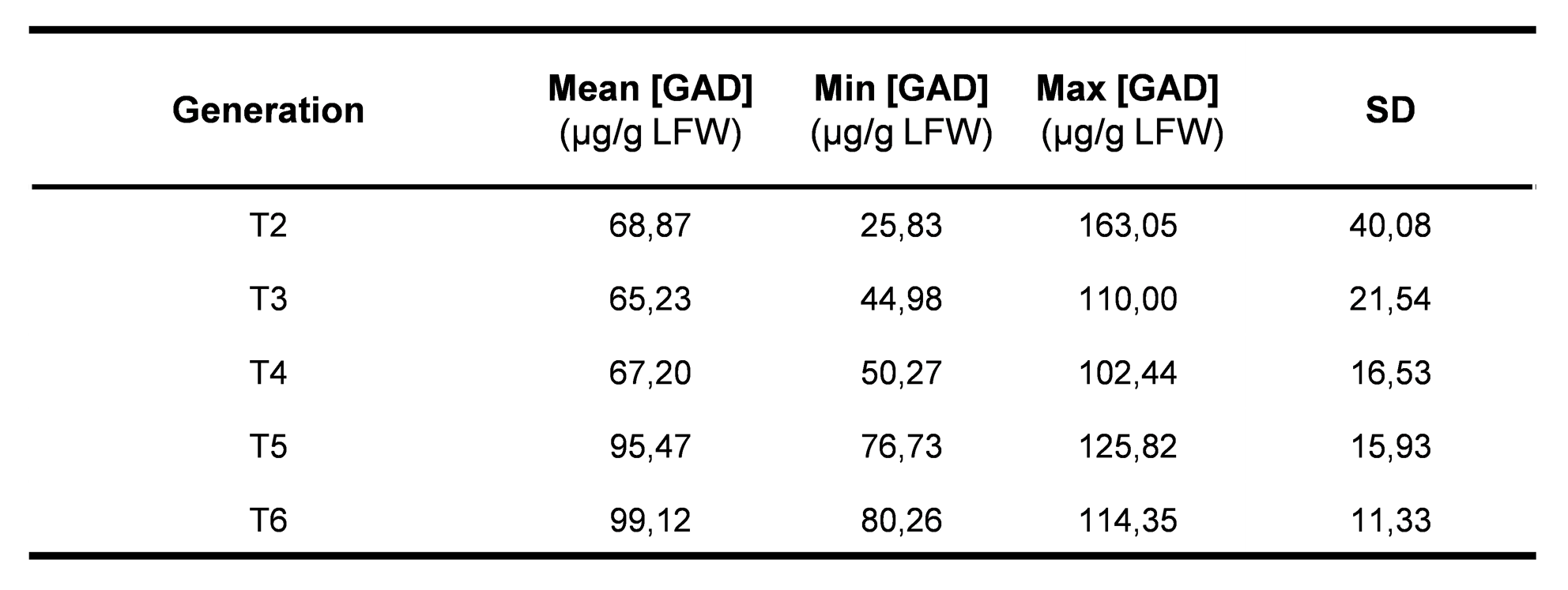

Supplement: Supplementary file 1 — Online Resource 1 (Table) Mean, maximum and minimum accumulation values are reported as µg hGAD65mut/g FLW calculated from radioimmunoassay data, and corresponding standard deviations. (TIFF 4760 kb) [file 11248_2013_9749_MOESM1_ESM.tif]

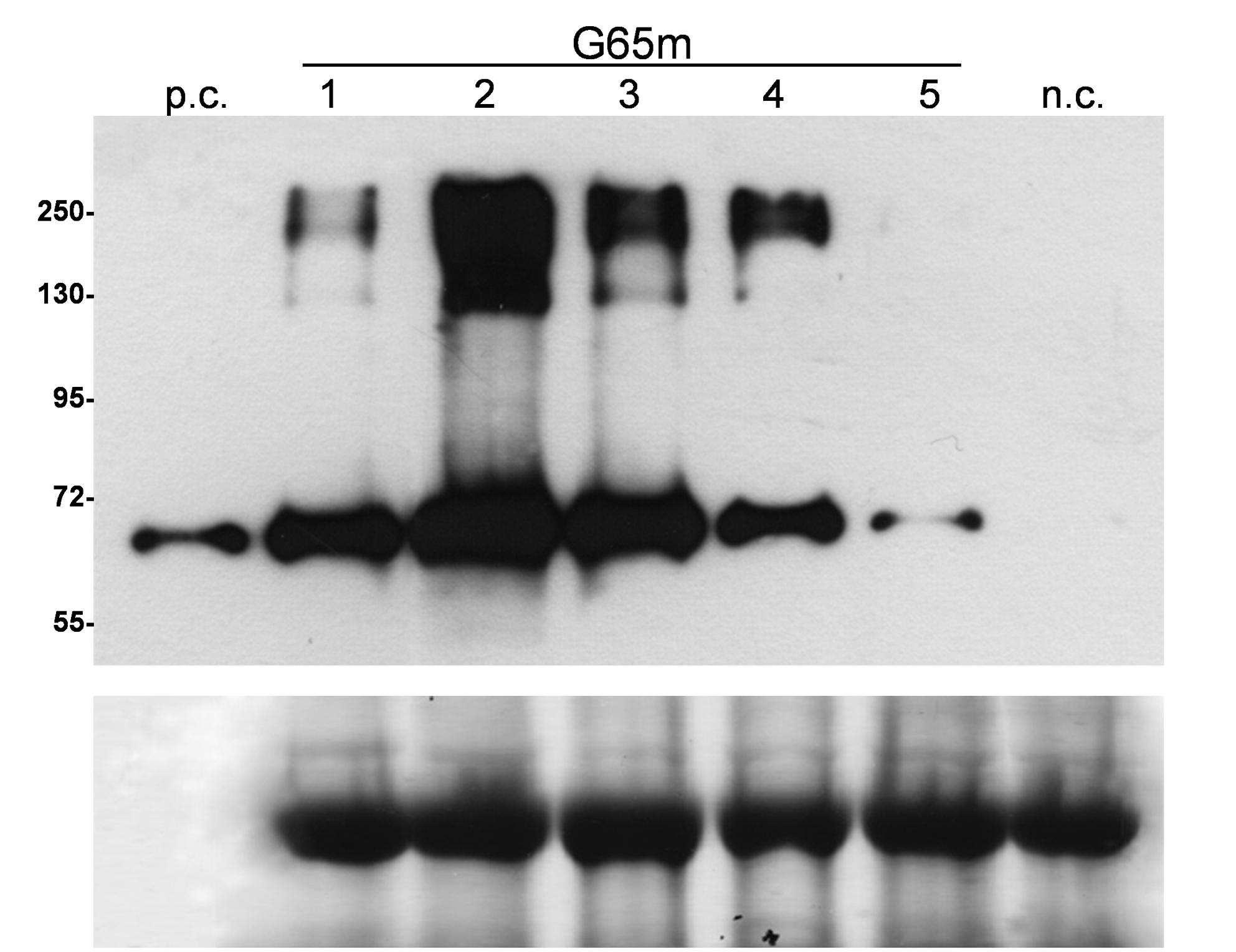

Supplement: Supplementary file 2 — Online Resource 2 (Figure) Transient expression of hGAD65mut in Nicotiana benthamiana plants using the pK7WG2 vector. Samples were collected daily from the 1-5 days post infiltration (dpi) (lanes 1-5). First panel, western blot of hGAD65mut (G65m) in leaf extracts (2.5 µg TSP per lane) detected using the GC3108 antibody. Second panel, loading control stained with Coomassie Brilliant Blue. Numbers indicate the molecular mass markers in kDa. n.c.= negative control, plants infiltrated with the pK7WG2 vector carrying the gfp marker gene; p.c.=positive control, 10 ng of commercial rhGAD65-His6 produced in the baculovirus/insect cell system. (TIFF 14749 kb) [file 11248_2013_9749_MOESM2_ESM.tif]

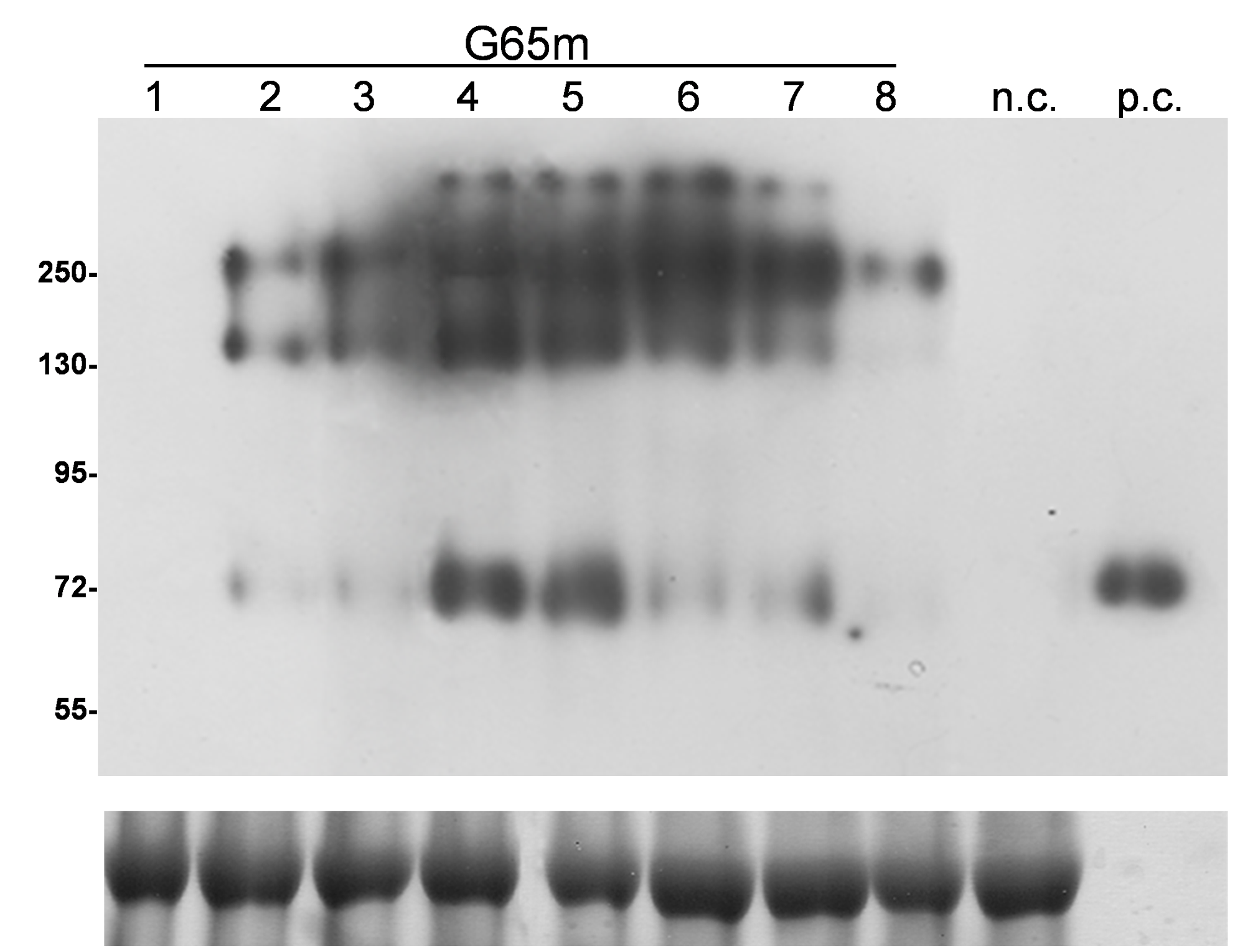

Supplement: Supplementary file 3 — Online Resource 3 (Figure) Transient expression of hGAD65mut in Nicotiana benthamiana plants using MagnICON vectors. Samples were collected daily from 1–8 days post infiltration (dpi) (lanes 1–8). First panel, western blot of hGAD65mut (G65m) in leaf extracts (5 µg TSP per lane) detected using the GC3108 antibody. Second panel, loading control stained with Coomassie Brilliant Blue. Numbers indicate the molecular mass markers in kDa. n.c. = negative control, plant infiltrated with the pICH20111 5’-module and pICH14011 integrase-module; p.c. = positive control, 10 ng of commercial rhGAD65-His6 produced in the baculovirus/insect cell system. (TIFF 12732 kb) [file 11248_2013_9749_MOESM3_ESM.tif]

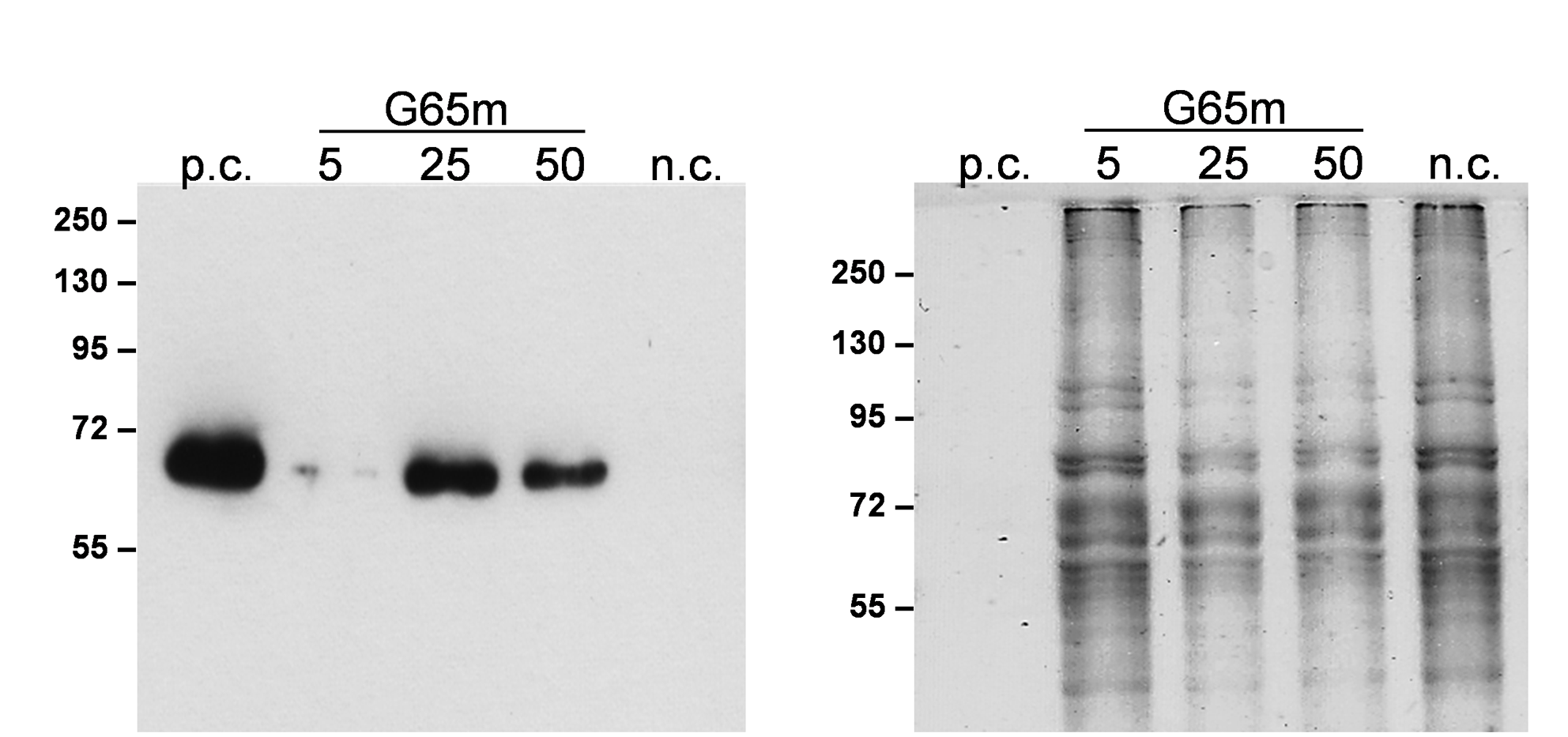

Supplement: Supplementary file 4 — Online Resource 4 (Figure) Expression of hGAD65mut using the baculovirus/insect cell platform. The following viral stock volumes were tested: 5, 25 and 50 µl. Left panel, western blot of hGAD65mut (G65m) in cell extracts (5 µg TSP per lane). Right panel, loading control stained with Coomassie Brilliant Blue. Numbers indicate the molecular mass markers in kDa. n.c. = negative control, extract of non-transformed insect cells; p.c. = positive control, 10 ng of commercial rhGAD65-His6 produced in the baculovirus/insect cell system. (TIFF 8599 kb) [file 11248_2013_9749_MOESM4_ESM.tif]

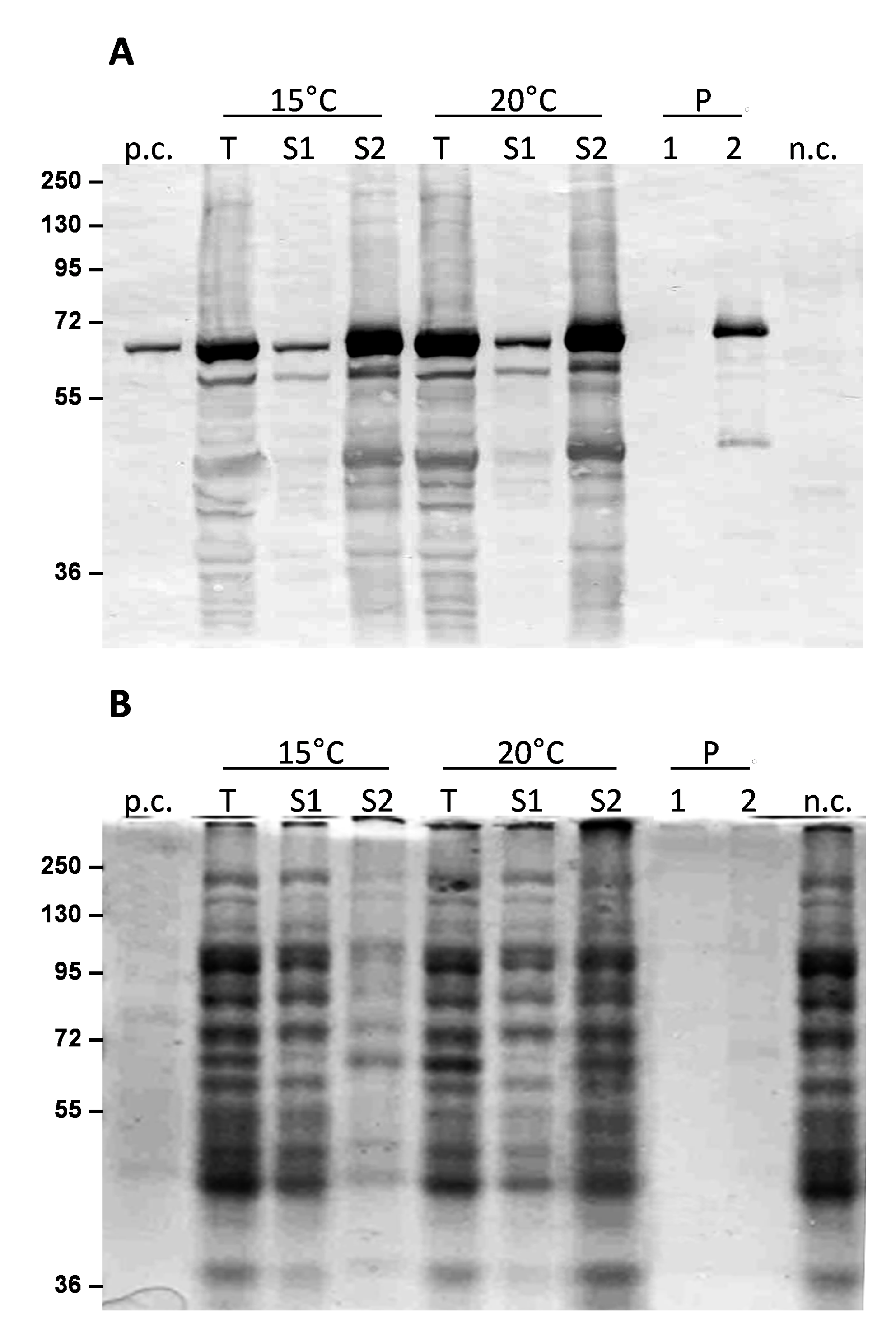

Supplement: Supplementary file 5 — Online Resource 5 (Figure) Expression of hGAD65mut using the Escherichia coli inducible expression platform. Bacterial cells were grown at 15°C or at 20°C. Upper panel, western blot of hGAD65mut in cell extracts (2 µg TSP per lane). Lower panel, loading control stained with Coomassie Brilliant Blue. Numbers indicate the molecular mass markers in kDa. n.c. = negative control, bacterial cells transformed with the pDEST17 ‘empty’ vector; p.c. = positive control, 15 ng of commercial rhGAD65-His6 produced in the baculovirus/insect cell system; T: Total samples; S1: Supernatant after sonication and centrifugation; S2 and P: Supernatant and pellet after centrifugation of the sample solubilized in urea-containing buffer. (TIFF 23553 kb) [file 11248_2013_9749_MOESM5_ESM.tif]
